# Effects of developmental and adult environments on ageing

**DOI:** 10.1111/evo.14567

**Published:** 2022-07-19

**Authors:** Krish Sanghvi, Maider Iglesias‐Carrasco, Felix Zajitschek, Loeske E. B. Kruuk, Megan L. Head

**Affiliations:** ^1^ Reserach School of Biology Australian National University Canberra ACT 2601 Australia; ^2^ School of Biology Earth and Environmental Sciences University of New South Wales Sydney NSW 2052 Australia

**Keywords:** Beneficial acclimation, *Callosobruchus maculatus*, developmental stress, matching environments, phenotypic plasticity, senescence

## Abstract

Developmental and adult environments can interact in complex ways to influence the fitness of individuals. Most studies investigating effects of the environment on fitness focus on environments experienced and traits expressed at a single point in an organism's life. However, environments vary with time, so the effects of the environments that organisms experience at different ages may interact to affect how traits change throughout life. Here, we test whether thermal stress experienced during development leads individuals to cope better with thermal stress as adults. We manipulated temperature during both development and adulthood and measured a range of life‐history traits, including senescence, in male and female seed beetles (*Callosobruchus maculatus*). We found that thermal stress during development reduced adult reproductive performance of females. In contrast, life span and age‐dependent mortality were affected more by adult than developmental environments, with high adult temperatures decreasing longevity and increasing age‐dependent mortality. Aside from an interaction between developmental and adult environments to affect age‐dependent changes in male weight, we did not find any evidence of a beneficial acclimation response to developmental thermal stress. Overall, our results show that effects of developmental and adult environments can be both sex and trait specific, and that a full understanding of how environments interact to affect fitness and ageing requires the integrated study of conditions experienced during different stages of ontogeny.

Early life conditions can act directly on developing phenotypes and in consequence can have both immediate and long‐lasting effects on a range of fitness‐related traits (van de Pol et al, 2006; Frankenhuis et al, 2019). For example, various studies have shown that individuals that experience a favorable developmental environment have increased performance as adults compared to individuals that experience poor developmental conditions (e.g., Descamps et al, 2008; Grafen, 1988; Klepsatel et al, 2020, 2019; Madsen and Shine, 2000; Monaghan, 2008; Müller et al., 2016; Sanghvi et al, 2021; Wong and Kölliker, 2014). These responses can sometimes be due to changes in developmental resources, as seen in the “silver‐spoon effect” (Grafen, 1988; Monaghan, 2008), or could be due to changes in developmental stress (henceforth referred to as “developmental stress response”).

The effects of developmental environments on fitness are also expected to depend on conditions experienced during adulthood (Gluckman et al, 2005, Monaghan, 2008). It has been suggested that individuals experiencing certain conditions during development may adjust their phenotype to improve performance when exposed to the same conditions as adults (“environmental matching” or “predictive adaptive response”) (Bateson et al, 2014). Under this scenario, developmental conditions shape the phenotype in response to predicted adult conditions, so that fitness is maximized when environments experienced during development and adulthood match (Bateson et al, 2014; Beaman et al, 2016; Cleal et al, 2007; Hayward and Lummaa, 2013). A special case of the environmental matching hypothesis, the “beneficial acclimation” response (Huey et al, 1999; Woods and Harrison, 2002), deals explicitly with stressful developmental and adult temperatures. It predicts that stressful temperatures experienced during development acclimate individuals such that they perform better when they also experience these stressful temperatures as adults, compared to individuals that only experience thermal stress as adults and not during development (e.g., Bahrndorff et al, 2016; Deere and Chown, 2006; Kellermann et al, 2017; Kristensen et al., 2008; Scharf, Galkin et al, 2015; Scott and Johnston, 2012). Another hypothesis that predicts that adult environments are important for determining later life fitness consequences of developmental environments is the “environmental saturation” hypothesis (Engqvist and Reinhold 2016; Pigeon et al, 2019). This hypothesis predicts that in favorable adult environments, all individuals will perform well regardless of their developmental environment, and likewise, that all individuals will perform poorly in bad adult environments. Thus, effects of developmental environments on adult phenotypes are only evident in intermediate adult environments (e.g., Pigeon et al, 2019).

Empirical evidence from studies considering how developmental and adult environments interact to affect adult traits is not clear cut, and indicates that the various hypotheses invoked to explain the relationships between phenotypic and environmental variation are not mutually exclusive (Pigeon et al, 2019). For instance, some studies find evidence for beneficial environmental matching (Duxbury and Chapman, 2020), whereas others find other types of interactive (Briga et al, 2017), or only additive (Kleinteich et al, 2015), effects of exposure to poor conditions during both development and adulthood. Additional complexity to the tangled interactions between developmental and adult life conditions arises from the fact that responses to environmental stimuli can be trait and sex dependent (e.g., Duxbury and Chapman, 2020; Helle et al, 2012; Krause et al, 2017; Min et al, 2020; Pigeon et al, 2019; Santos et al, 2021; Scharf, Braf et al, 2015; Stillwell and Fox, 2005). For example, in cichlids reproductive rate is determined only by nutrition during development, whereas adult growth rate is determined only by nutrition in the adult stage, and clutch size is determined by both developmental and adult life nutrition (Taborsky, 2006). Differences in the way the environment affects different traits may result from energetic and physiological constraints acting on life‐history traits (Partridge and Silby 1991), as well as the necessity to allocate resources across traits. Furthermore, differences between males and females in their life‐histories and mating strategies mean that selection might favor males and females to respond differently to the same environment (Ceballos and Valenzuela 2011; Maklakov et al, 2009; Stillwell et al. 2010), possibly by selecting for sex‐specific pace of life syndromes (Immonen et al, 2018). For example, in seed beetles, males and females respond differently to the presence and density of competitors during the larval stage, leading to sex‐specific differences in a variety of life‐history traits (Iglesias‐Carrasco et al 2020; Sanghvi et al, 2021).

Research shows that in addition to influencing the absolute expression of traits, the environment can also influence how traits change over an individual's life, and specifically how they deteriorate with advancing age (i.e., how they senesce) (Balbontín and Møller, 2015; Nussey et al, 2007). Senescence occurs as a consequence of relaxed selection on fitness‐related traits in older individuals due to trade‐offs between life‐history components (Rose and Charlesworth, 1980; Stearns, 1989). However, the rate at which individuals age may depend on a range of factors such as their sex and external environment (e.g., Sanghvi et al, 2021). Although there is some support for favorable developmental conditions leading to slower reproductive and survival senescence (Hayward, Wilson et al, 2013, Cooper and Kruuk, 2018, Sanghvi et al, 2021), an alternative hypothesis suggests that individuals experiencing good environments may senesce faster due to increased investment in growth and reproduction when young (Adler et al, 2016; Hooper et al 2017; Hunt et al, 2004; Spagopoulou et al., 2020). Additionally, senescence can also depend on the interactions between developmental and adult environments, as seen in studies that test for compensatory growth. Here, organisms that experience poor developmental environments increase their investment in growth in favorable adult environments, although at the cost of increased mortality (Dmitriew and Rowe, 2007; Metcalfe and Monaghan, 2001). Although recent research has begun investigating how interactions between developmental and adult environments affect survival and reproductive senescence (Duxbury and Chapman, 2020; Min et al, 2020; Zajitschek et al, 2009), the results do not clearly support one hypothesis (developmental stress response, beneficial acclimation, or environmental saturation).

Here, we test for interactions between effects of heat stress experienced during development and adulthood on life‐history traits in male and female seed beetles (*Callosobruchus maculatus*). In seed beetles, hot developmental temperatures have been shown to reduce larval survival, emergence weight, and testes size (Fox et al, 2011; Stillwell and Fox, 2005; Stillwell and Fox, 2007; Stillwell et al, 2007; Vasudeva et al, 2014), whereas hot adult temperatures have been shown to reduce female fecundity and overall fitness (Stillwell and Fox, 2005). Despite these widespread effects, the exact mechanism by which thermal environments experienced at different stages interact to affect age‐dependent reproduction, survival, and weight in seed beetles is unknown. We measured survival in both sexes, reproductive success in females, and weight in males, and tested for senescence in each of these. We considered weight in males over more direct measures of reproductive fitness (which are also more difficult to obtain) because weight is highly correlated with a range of fitness traits in seed beetle males, such as ejaculate size (Savalli and Fox, 1999; Vasudeva et al, 2014) and survival (Tatar et al, 1993).

Temperature is known to be crucial in determining life‐history traits, including senescence, and physiology of ectotherms (Zuo et al, 2012) and is often manipulated in studies that test for effects of stressful developmental conditions (e.g., Scharf, Braf et al, 2015), matching environments (e.g., Min et al, 2020), and beneficial acclimation (e.g., Geister and Fischer, 2007; Leroi et al, 1994). Studies like ours, which manipulate temperature at various life stages to understand phenotypic change, are crucial if we wish to model how climate change will affect life histories of animals. Because seed beetles are not obligated to eat or drink during adulthood (Beck and Blumer, 2014), it makes them a model species for taxa that cannot compensate for a stressful developmental environment by feeding or drinking more during adulthood. High temperatures can desiccate insects; thus, such desiccating effects of thermal stress would be exacerbated in species such as seed beetles that do not drink as adults.

## Methods

### ORIGIN AND MAINTENANCE OF STUDY POPULATION

Our stock population of *C. maculatus* was sourced in 2017 from stock kept at the University of Western Australia (see Dougherty et al. [2017] for maintenance details). Once in our lab, stock was maintained for 14–16 generations on cowpea beans (*Vigna unguiculata*) at 24–28°C and 20%–40% relative humidity. Neither stock nor experimental beetles were provided with food or water as adults, as is the norm for seed beetle experiments.

### EXPERIMENTAL DESIGN

To test the effects of developmental and adult temperatures on life‐history traits and senescence, we used a split‐brood full‐sib 2 × 2 factorial design in which beetles were assigned to either “ancestral” (i.e., relatively cooler) temperatures (23–25°C) or “hot” temperatures (33–36°C) during development, and then ancestral or hot temperatures as adults. The ancestral temperature was at the lower end of the temperature range in which the stock had been raised for over 14–16 generations. The hot temperature was a novel and unfavorable environment (see *Results*) for this population.

To breed experimental beetles, we collected 86 male and 86 female virgin seed beetles from 150 isolated stock beans, within an hour from when they emerged. Virgin females were randomly paired with virgin males for mating and then given 20–30 beans on which to lay eggs (at 24°C), so that each parental pair contributed a maximum of 30 offspring to our experiment (see Supporting Information A). Seed beetle females can lay many more than 30 eggs per day (Sanghvi et al, 2021), thus limiting their eggs laying in early life could possibly affect their late‐life offspring. Although, because in our study, we only collected offspring from parental females on the first 2 days of eggs laying, we do not think that limiting egg laying of parental females would impact our results. We checked beans for eggs every few hours in the day and removed beans from the petri dish whenever they had an egg laid on them. Extra eggs (i.e., >1 egg per bean), when present, were scraped off prior to hatching and it was ensured visually during emergence that each bean had only one beetle developing in it. Beans with a single egg laid on them were transferred to individual Eppendorf tubes and then randomly assigned to a hot (33–36°C) or to an ancestral (23–25°C) developmental temperature for incubation until adults emerged. On the day of emergence, beetles were weighed (to the nearest 0.01 mg) using a Sartorius Cubis microbalance, and their developmental time (in days) and sex were recorded. Beetles were then assigned to either the hot or ancestral adult temperature treatment, in which they remained until they died. This generated four treatments: *ancestral developmental and ancestral adult* (AA), *ancestral developmental and hot adult* (AH), *hot developmental and ancestral adult* (HA), and *hot developmental and hot adult* (HH) temperatures (see Table S1 for sample sizes).

Experimental males were kept in their Eppendorf tubes and weighed every second day. Experimental females were individually mated with a single male from the stock population on the day of their emergence, irrespective of their emergence durations. To ensure that females throughout our experiment mated (rather than just being mounted but not copulated by a male), we observed whether the female kicked the male with her hind legs to end copulation (Berger et al, 2016; Wilson and Tomkins, 2014). If she did not, we paired her with the same male again after ∼20 min, after which all females copulated. After copulation, females were then transferred to a Petri dish and given 15 new beans each day to lay eggs on. Beans with eggs laid on them were stored in plastic bags, and frozen the next day, at –20°C for counting later. Both sexes were checked daily for survival and their adult life span was recorded. The experiment was conducted over three experimental blocks run at different times (Block 1: 26 families; Blocks 2 and 3: 30 families each). The individuals used in the three experimental blocks came from three successive generations of stock beetles (i.e., blocks 1, 2, and 3, correspond to generations 14, 15, and 16, respectively). Some experimental beetles escaped, were killed accidentally, or could not have their sex identified accurately during the experiment, and were therefore excluded from analyses of reproduction, weight, and life span (57 excluded out of 1381 beetles). The assignment of beetles to the developmental and adult treatments was done such that each sex from each of the 86 families was approximately evenly distributed across the four treatments. The observer was blinded to the treatment of beetles during data collection to avoid bias.

We collected data on the following age‐dependent traits: emergence success, development time, emergence weight, adult life span, female fertility, and female life span reproductive success. We also measured age‐dependent mortality of beetles, female age‐dependent (measured daily) fecundity, and male age‐dependent weight (measured every 2 days). Details and full definitions of all traits as well as details of how these traits were modeled are given in Table 1.

**Table 1:** Summary of traits and models used for analyses

Trait	Definition	Model Type	Error Distribution	Data Transformation (LMM)/Link Function (GLMM)	Fixed Effects	Random Effects
Emergence success	Likelihood of adults emerging from a bean with an egg laid on it	GLMM	Binomial	Logit	DevT. + Block	Family
Development time	Number of days between the laying of an egg and the emergence of an adult	LMM	Gaussian	Power ((*y^λ^* – 1)/*λ*) *λ* = –0.14141	Sex × DevT. + Block	Family
Emergence weight	Weight (mg) of beetle on the day of emergence	LMM	Gaussian		Sex × DevT. + Block	Family
Adult life span	Number of days between the emergence of an individual and its death	LMM	Gaussian		Sex × DevT. × AdultT. + Emergence weight + Block	Family
Fertility (females)	Likelihood that a female laid at least one egg in her lifetime	GLM	Binomial	Logit	DevT. × AdultT. + Block	None
Life time reproductive success (females)	LRS: total number of eggs laid by a female in her lifetime	GLMM	Poisson	Log_e (*y*)	DevT. × AdultT. + Adult Life span + Block	Family, observation
Age dependent (daily) fecundity (females)	Number of eggs laid by a female each day, measured since day of emergence throughout her life span	GLMM	Negative binomial distribution	Log_e (*y*)	DevT. × AdultT. × Age (and Age^2^) + Adult Life Span + Block	Age|Family and Age|ID as random effects)
Age‐dependent weight (males)	Weight (mg) of males measured every alternate day, since day of emergence, throughout his life span	LMM	Gaussian	None	DevT. × AdultT. × Age (and Age^2^) + Age × Emergence weight + Adult Life Span + Block	Family, Individual ID
Age‐dependent survival	Likelihood of adult beetles dying at a given adult age	Cox proportional hazards		None	Sex × DevT. × AdultT. + Block	Family

Abbreviations: AdultT. = adult temperature; Age = adult age; DevT. = developmental temperature.

### DATA ANALYSIS

To determine the effects of developmental and adult temperature as well as their interaction on the life‐history traits including senescence, we used Generalised Linear Mixed‐effects Models (GLMM) or Linear Mixed‐effects Models (LMM) as appropriate. All analyses were conducted in R version 3.5.2 (R Development Core Team, 2011) and models were built using the *lme4* (Bates et al, 2015), *coxme* (Therneau, 2014), and *glmmTMB* (Magnusson et al, 2017) packages. Model details for each trait are given in Table 1 and in Section B of the Supporting Information. All models contained experimental block as a three‐level fixed effect and beetle family (i.e., full‐sibling groups) as a random effect unless mentioned otherwise.

For traits measured prior to the assignment of adult treatments (i.e., emergence success, development time, and emergence weight), we included developmental temperature in the model as a fixed effect. For traits measured after beetles were assigned to adult temperatures (i.e., adult life span, female fertility, female lifetime reproductive success [LRS], age‐dependent [daily] female fecundity, age‐dependent male weight, and age‐dependent mortality), we included both developmental and adult temperatures as fixed effects. For all traits that were measured on both sexes (i.e., development time, emergence weight, adult life span, age‐dependent mortality), we included sex and its interaction with the temperature variables as a fixed effect to allow us to test for sex‐specific responses (Garcia‐Sifuentes and Maney, 2021). For traits measured at multiple ages (i.e., age‐dependent female fecundity and age‐dependent male weight), the age (days) at which the measurements were taken was included as a fixed effect (covariate).

For age‐dependent female fecundity and age‐dependent male weight, we additionally included each individual's adult life span as a fixed effect in the models to account for selective disappearance (van de Pol and Verhulst, 2006). Further, for male and female life span we included emergence weight as a covariate because larger seed beetles have been previously shown to live longer (Fox et al, 2003). For age‐dependent weight of males, emergence weight was also included as an interaction with age, because males at different ages could be affected by their emergence weight in different ways. For models where repeated measurements were made on the same individual, that is, age‐dependent female fecundity and age‐dependent male weight, we added a random effect of beetle ID to avoid pseudoreplication.

In general, we started with a “full model” that included two‐ and three‐way interactions between developmental temperature, adult temperature, sex, and age. Specifically, in the full model, three‐way interactions were fit whenever developmental and adult temperatures interacted either with sex (to test for sex‐specific effects) or age (to test for effects on senescence), whereas two‐way interactions were fit whenever developmental temperatures interacted with sex. These “full models” were used to interpret the highest order level interactions (three‐ or two‐way, depending on the fixed effects included). To then interpret lower order interactions (i.e., two‐way interactions when the full model had three‐way interactions) and the main effects of fixed effects, we fitted models with the higher order level interactions removed (i.e., a model with just two‐way interactions and main effects or a model with just main effects, respectively). This was done so that parameter estimates would reflect the overall influence of these effects, averaged across all levels of other variables (Engqvist, 2005).

We modeled female age‐dependent (daily) fecundity with a negative binomial error distribution (using package *glmmTMB*) because females did not lay any eggs on most days. This model fitted random intercepts of different females and different families and random slopes to allow the effects of age to vary between families and females (henceforth called “Global model”). To test for the presence of (broad‐sense) heritability in female age‐dependent fecundity, we compared this “Global model” to a model without any random effects of family (either intercepts or slopes) using Akaike Information Criterion (AIC) and log‐likelihood ratio tests with the *anova* function in the *stats* package.

We explored the effects of age and environment on male weight further, for males that experienced hot adult temperatures. This was done to test whether males from AH treatments had a faster rate of age‐dependent weight loss than males from HH treatments, which would possibly indicate a beneficial acclimation effect.

For all linear models, residuals were checked visually to ensure they met assumptions of normality and homoscedasticity. When they did not, the response variable was transformed (see Table 1). To test for overdispersion in our models, we used the function *simulateResiduals* in the package *DHARMa* (Hartig 2020). There was evidence for overdispersion (in female LRS), so we additionally fitted an observation‐level random effect (Harrison 2014). Wherever appropriate, effect sizes were calculated as “Hedge's *g*” for all two‐group comparisons (following eqs. 1 and 2 in Nakagawa and Cuthill, 2007) to indicate the strength of the effect seen. Given the large number of tests performed in the study, we set the critical α level for *P*‐values to 0.01 instead of 0.05.

## Results

Our analyses indicated a range of effects of developmental and adult temperature on seed beetle traits. We describe these results below, and give full output for each model in Tables S3–S12.

### AGE‐INDEPENDENT TRAITS

#### Emergence rate

Hot developmental temperature reduced the emergence success of beetles (*z* = –14.853, *P* < 0.001; Table S3): 78% of eggs emerged as adult beetles from the ancestral developmental temperature, whereas 49% emerged as adult beetles from the hot developmental temperature.

#### Developmental time

Hot developmental temperatures accelerated developmental times of beetles. Specifically, beetles that survived to emergence that had experienced hot developmental temperatures had a shorter development time than those experiencing ancestral developmental temperatures (DF = 1337, *t* = –94.522, *P* < 0.001; Table S4), with both males and females being affected in a similar way (DF = 1337, *t* = –0.731, *P* = 0.465; Table S4) (mean ± SE for Hot developmental temperatures: males = 23.1 ± 0.1, females = 23.5 ± 0.2 days; Ancestral developmental temperatures: males = 37.8 ± 0.2, females = 37.99 ± 0.2 days; Hedge's *g*: males = 4.41, females = 4.962).

#### Emergence weight

Overall, hot developmental temperatures lead to the emergence of lighter beetles. There was a significant interaction of developmental temperature and sex on the emergence weight of beetles (DF = 1327, *t* = 12.710, *P* < 0.001; Table S5), whereas on average, beetles showed a decrease in emergence weight when developing in hot temperature (DF = 1328, *t* = –18.837, *P* < 0.001; Table S5): this reduction in weight was more severe for females than for males (Fig. S1; Hedge's *g*: males = 0.436, females = 1.4) (mean ± SE for males: 3.510 ± 0.030 in hot developmental, and 3.750 ± 0.030 in ancestral developmental temperatures; for females: 4.710 ± 0.039 mg in hot developmental, and 5.760 ± 0.042 mg in ancestral developmental temperatures).

#### Adult life span

**Figure 1:**
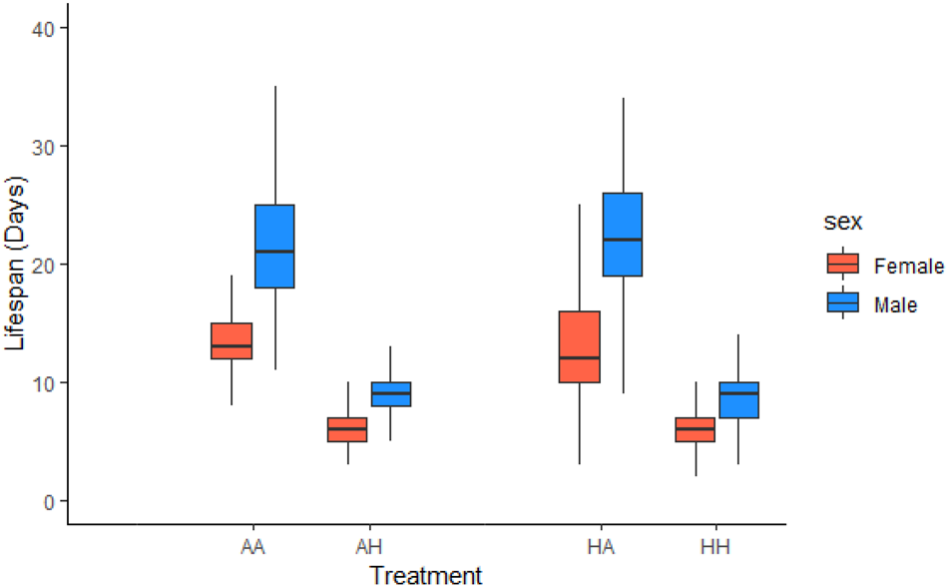
The effects of developmental and adult temperatures on female (red) and male (blue) adult life span (raw data): *ancestral developmental and ancestral adult* (AA), *ancestral developmental and hot adult* (AH), *hot developmental and hot adult* (HH), and *hot developmental and ancestral adult* (HA) temperatures. Median, interquartile ranges (5%, 25%,50%, 75%, and 95%) presented.

Hot developmental and hot adult temperatures had contrasting effects on adult life span. Overall, adult life span was not affected by the three‐way interaction between developmental temperature, adult temperature, and sex (DF = 1253, *t* = –0.691, *P* = 0.490; Table S6; Fig. 1). Nor was it affected by the two‐way interaction between developmental and adult temperature (DF = 1254, *t* = –1.297, *P* = 0.195) or between developmental temperature and sex (DF = 1298, *t* = –1.283, *P* = 0.2). However, there was a significant effect of the interaction between adult temperature and sex on life span (DF = 1251, *t* = –14.781, *P* < 0.001; Table S6). Specifically, hot adult temperatures on average decreased life span of beetles compared to ancestral temperatures (DF = 1259, *t* = –51.097, *P* < 0.001), but this decrease was greater for males than for females (Fig. S2; Males: Hedge's *g* = 4.820; Females: Hedge's *g* = 2.493). In contrast, hot developmental temperatures on average increased adult life span of beetles compared to ancestral developmental temperatures (DF = 1304, *t* = 4.3, *P* < 0.001; Table S6; Males: Hedge's *g* = 0.10; Females: Hedge's *g* = 0.06).

#### Female fertility

Females that developed in hot temperatures were less likely to be fertile than females that developed in ancestral temperatures (15.5% of females from the hot developmental temperature did not lay any eggs compared to 0.6% of females from the ancestral developmental temperature, *z* = –5.405, *P* < 0.001; Table S7). Neither adult temperature on its own (*z* = –1.557, *P* = 0.119) nor its interaction with developmental temperature (*z* = 0.017, *P* = 0.986) had a significant effect on female fertility.

#### Female LRS

**Figure 2:**
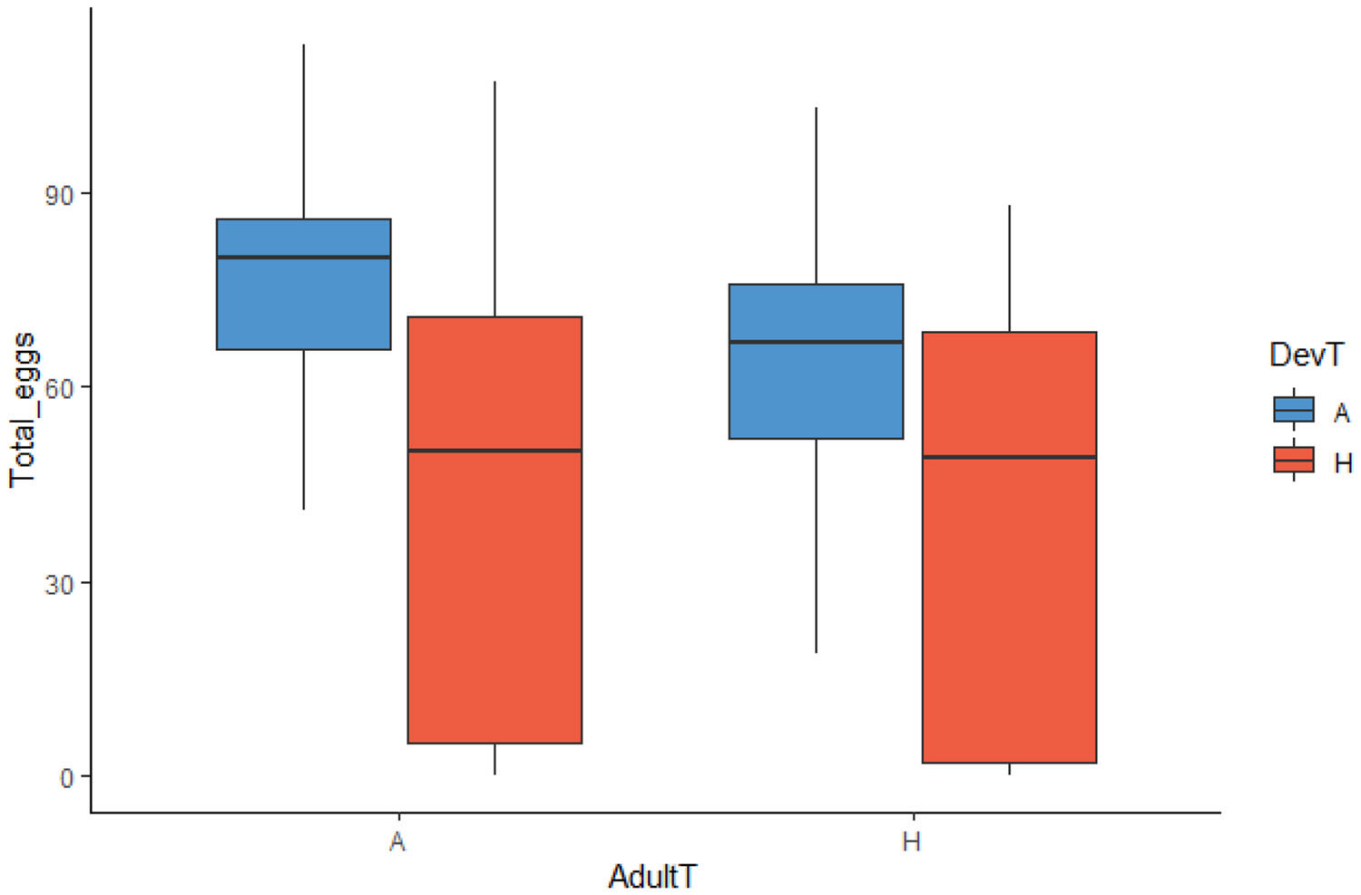
Box plot comparing the median, interquartile ranges (5%, 25%, 50%, 75%, and 95%) of the effects of developmental (DevT) and adult temperature (AdultT) on female lifetime reproductive success (Total_eggs). H = hot temperature; A = ancestral temperature.

In general, there were additive effects of hot developmental and hot adult temperatures on female LRS. There was no effect of the interaction between developmental and adult temperatures on female LRS (*z* = –1.637, *P* = 0.102). However, both hot developmental (*z* = –16.223, *P* < 0.001, Hedge's g = 0.945) and hot adult (*z* = –10.366, *P* < 0.001, Hedge's g = 0.241) temperatures independently reduced the LRS of females, compared to ancestral developmental and adult temperatures, respectively (Fig. 2; Table S8).

### AGE‐DEPENDENT TRAITS

#### Age‐dependent mortality

**Figure 3:**
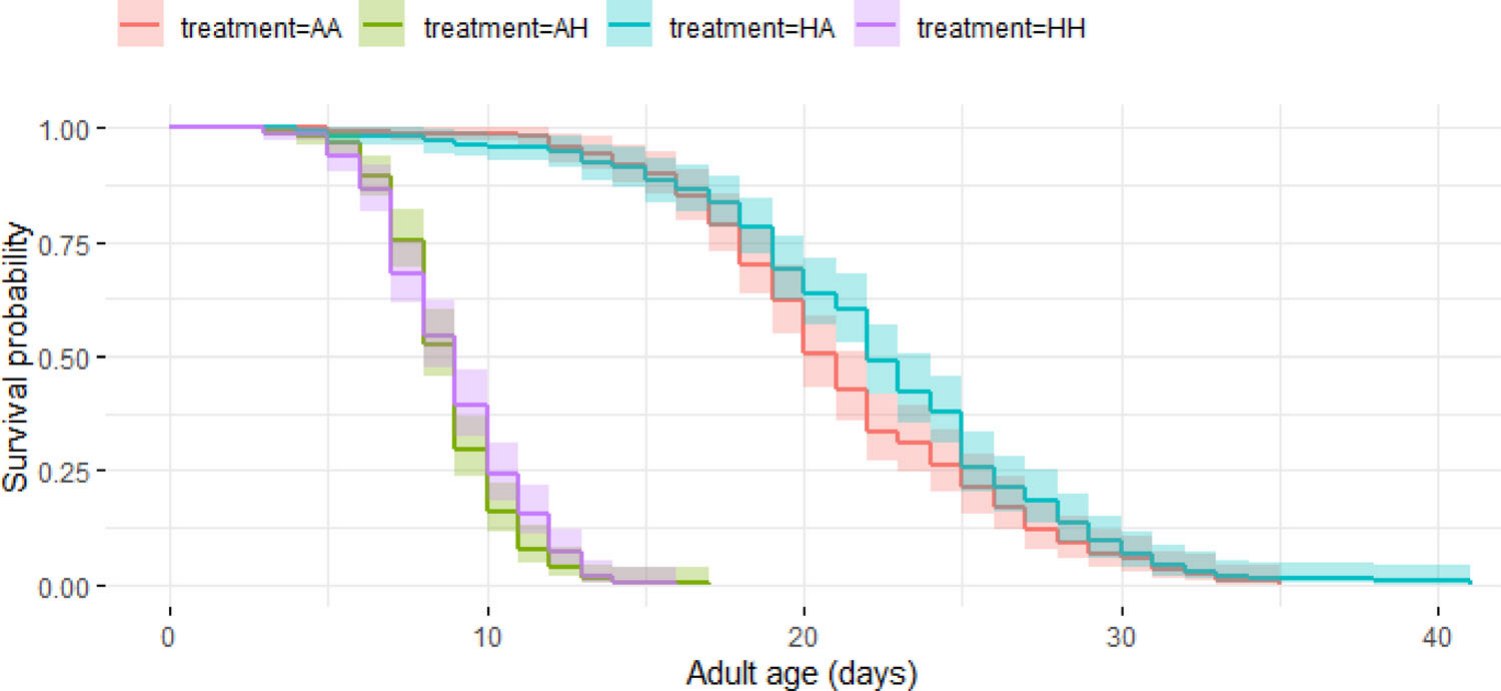
The survival probability of adult males from four treatments with increasing age, namely: *ancestral developmental and ancestral adult* (AA‐red), *ancestral developmental and hot adult* (AH‐green), *hot developmental and hot adult* (HH‐purple), and *hot developmental and ancestral adult* (HA‐blue) temperatures, using Kaplan‐Meier curves. Shaded regions represent 95% confidence intervals.

**Figure 4:**
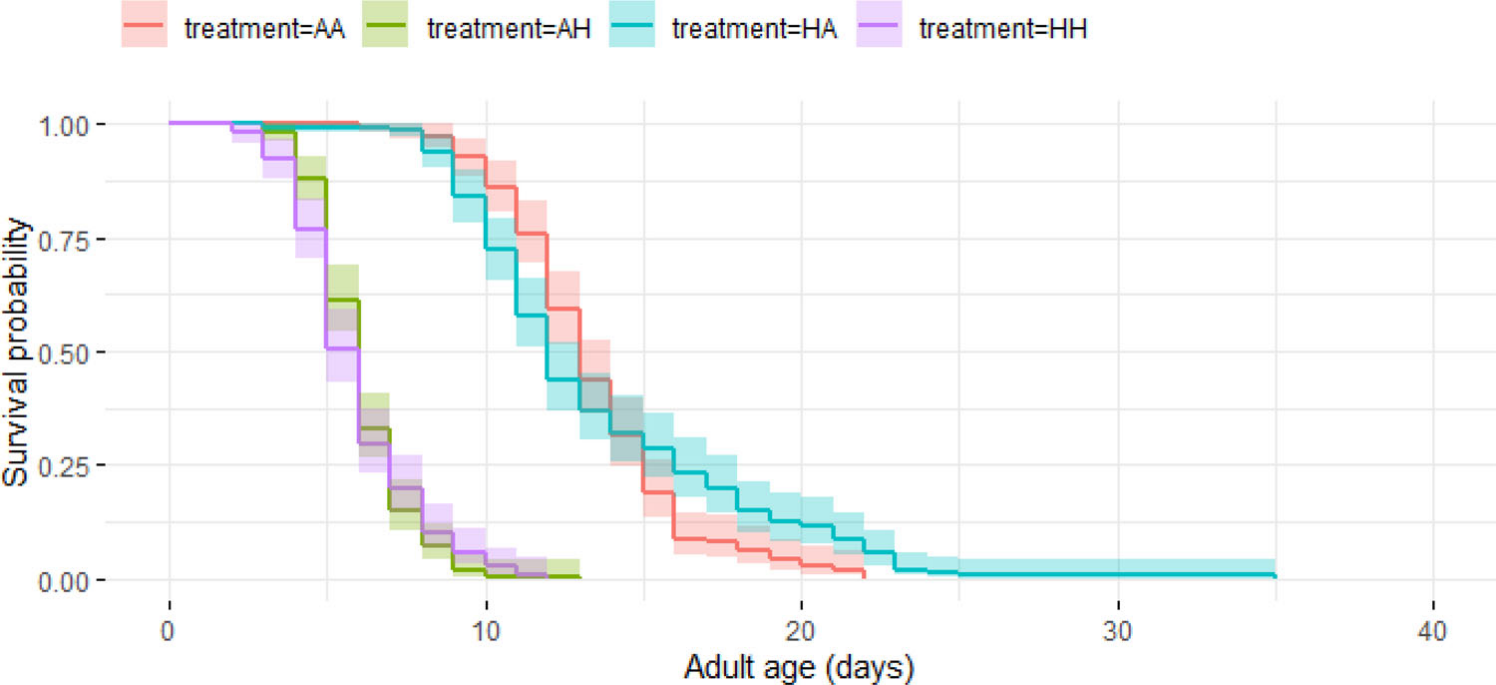
The survival probability of adult females from four treatments with increasing age, namely: *ancestral developmental and ancestral adult* (AA‐red), *ancestral developmental and hot adult* (AH‐green), *hot developmental and hot adult* (HH‐purple), and *hot developmental and ancestral adult* (HA‐blue) temperatures, using Kaplan‐Meier curves. Shaded regions represent 95% confidence intervals.

In general, hot developmental and hot adult temperatures had contrasting effects on age‐dependent mortality of beetles. Age‐dependent mortality was not affected by any of the three‐ or two‐way interactions between sex, developmental temperature, and adult temperature (–1.3 < *z* < 1.95, all *P*‐values >0.05; Table S9; Figs. 3 and 4). Similar to the effects of hot developmental temperature seen on adult life span, beetles that developed in hot temperatures were more likely to have lower age‐dependent mortality (i.e., slower rate of survival senescence) than beetles from ancestral developmental temperatures (*z* = –3.03, *P* = 0.002). In contrast, beetles that experienced hot adult temperatures had a faster rate of age‐dependent mortality than beetles from ancestral adult temperatures (*z* = 35, *P* < 0.001).

#### Age‐dependent (daily) female fecundity

**Figure 5:**
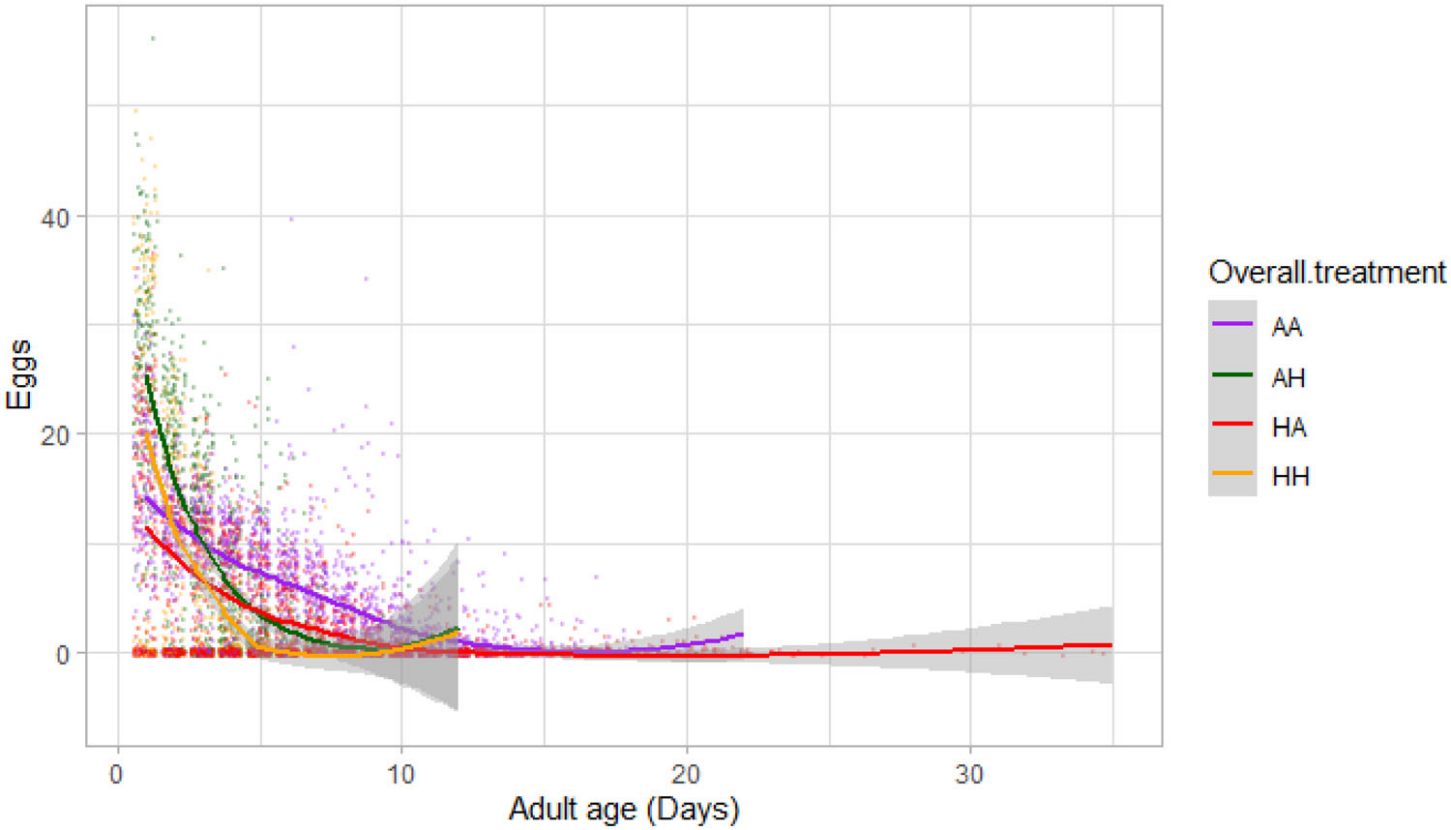
Effects of adult age on female daily fecundity for all four treatments: *ancestral developmental and ancestral adult* (AA‐Purple), *ancestral developmental and hot adult* (AH‐Green), *hot developmental and hot adult* (HH‐Orange), and *hot developmental and ancestral adult* (HA‐Red) temperatures. Shaded regions represent 95% confidence intervals. See Section D in the Supporting Information for an explanation for why females show increased average fecundity in late adult life.

In general, there were additive effects of hot developmental and hot adult environments on female reproductive senescence. Overall, age‐dependent female fecundity was not affected by the three‐way interaction between developmental temperature, adult temperature, and age (*z* = 0.097, *P* = 0.923) (Fig. 5; Table S10). However, developmental (*z* = –7.514, *P* < 0.001) and adult temperatures (*z* = –8.988, *P* < 0.001) each interacted with age to affect fecundity of females. Specifically, females that experienced either hot developmental or hot adult temperature showed a faster decline in fecundity with increasing age, compared to females that experienced ancestral developmental or ancestral adult temperatures, respectively. Although females in hot adult temperatures laid a higher number of eggs than females in ancestral adult temperatures in early adult life, the opposite was true for late adult life (Fig. 5). On the other hand, females from ancestral developmental temperatures always laid more eggs than females from hot developmental temperatures, when averaged across the effects of adult temperatures (Fig. 5). The model that allowed slopes and intercepts for age‐dependent (daily) fecundity to vary between females and between families (of full‐sibs) provided a better fit to the data than the model that did not have a random effect of family and only allowed the intercepts of different females to vary (ΔDF = 5, ΔAIC = 414). This suggests significant between‐family variation in female reproductive senescence rates.

#### Age‐dependent male‐weight

**Figure 6:**
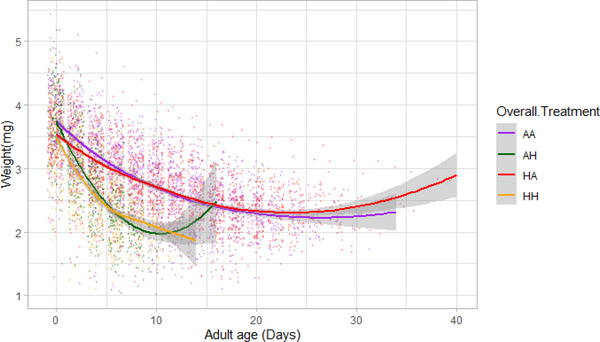
Effects of adult age on weight (mg) in males for all four treatments: *ancestral developmental and ancestral adult* (AA‐Purple), *ancestral developmental and hot adult* (AH‐Green), *hot developmental and hot adult* (HH‐Orange), and *hot developmental and ancestral adult* (HA‐Red) temperatures. Shaded regions represent 95% confidence intervals.

We found some evidence for a beneficial acclimation effect of hot developmental temperature on senescence of male weight. There was a significant effect of the three‐way interaction between developmental temperature, adult temperature, and age on male weight (DF = 4745, *t* = 5.1, *P* < 0.001; Table S11; Fig. 6). This interaction was due to males that experienced hot temperature during development and adulthood having a lower rate of age‐dependent weight decline than males that experienced hot temperatures during adulthood but ancestral developmental temperatures (DF = 1368, *t* = 7.011, *P* < 0.001; Table S12). When age‐dependent changes in weight were binned by life span (Fig. S3), they showed that heavier individuals lived longer and that male weight decreased throughout the lifetime of males (see Section D in the Supporting Information). This suggests that the apparent increase in average male weight seen in late adult life (evidenced by a significant quadratic effect of age in Table S11 and increase in weight toward the end of life in Fig. 6) is due to selective disappearance of lighter beetles (effect of life span: *P* < 0.001).

## Discussion

Environments experienced during development and adulthood can interact in complex ways to shape adult traits. Consequently, the results of studies testing the effects of exposure to favorable or unfavorable conditions both during the development and adulthood are mixed. To improve our understanding of how heat stress experienced at different life stages affects individual phenotypes, as well as to test whether interactive effects of developmental and adult‐life environments affect rates of ageing, we subjected juvenile and adult *C. maculatus* to a combination of stressful/hot temperatures and benign/ancestral temperatures. We then measured a range of age‐independent and age‐dependent traits in both males and females. We found that although female reproductive traits were affected negatively by stressful developmental temperature, nonreproductive traits, such as life span and age‐dependent mortality, were only affected negatively by stressful adult temperature. The only evidence for any interaction between developmental and adult environment was for male weight senescence. Below, we discuss the evidence for and against our two main hypotheses for how environments at different stages affect traits (developmental stress response and beneficial acclimation response). We then discuss the implications of other results we found, namely, sex‐specific effects and other general patterns in senescence.

### EVIDENCE FOR A RESPONSE TO STRESSFUL DEVELOPMENTAL ENVIRONMENTS

We found that individuals experiencing hot/stressful temperatures during development emerged sooner as adults than those experiencing ancestral temperatures. Such effects of developmental temperature on development time may inform us about how generation times and thus rates of evolution of ectotherms might change due to climate warming (Promislow et al, 2022). Development times of beetles in our study were on average longer compared to those seen in some (e.g., Berger et al., 2016) but not all studies of this species (e.g., Iglesias‐Carrasco et al, 2020; Sanghvi et al, 2021), possibly due to lab‐specific adaptations of these populations or differing levels of inbreeding. Accelerated development caused by high temperatures came with costs, such as lower body weight at emergence (which is well known to occur in ectotherms: Zuo et al, 2012), and lower emergence success. The lack of developmental stress response for adult life span and age‐dependent mortality could be due to hot developmental temperatures causing selective disappearance of poor‐quality larvae. This selection at the developmental stage would mean that only larvae of a higher quality would emerge as adults, thus masking the effects of temperature on adult survival.

In females, hot developmental temperatures resulted in reduced reproductive performance (lower LRS and fertility, faster reproductive senescence). This suggests a developmental stress response for female reproduction, where, independent of the adult environment, stressful developmental environments reduced female fitness (see also Cooper and Kruuk [2018] for review; Sanghvi et al, 2021). A possible explanation for this effect is that females experiencing hot developmental temperatures develop faster, emerge at a lower weight (Guntrip et al, 1997), and thus allocate more energy to somatic maintenance than to reproduction (Kirkwood and Austad, 2000; Maklakov and Chapman, 2019). Consequently, females experiencing hot developmental temperatures lay fewer eggs throughout their life, compared to females from ancestral developmental temperatures.

### EVIDENCE FOR A RESPONSE TO STRESSFUL ADULT ENVIRONMENTS

When exposed to hot temperatures as adults, females had higher early adult life reproduction but shorter life spans and faster reproductive senescence than females experiencing ancestral adult temperatures. These findings support classic life‐history theory (Partridge 1987; Stearns, 1989) that proposes a trade‐off between early adult and late adult life reproduction (Reed et al, 2008), and between survival and reproduction (Hammers et al, 2013; Kirkwood and Rose, 1991; Marshall et al, 2017). In the presence of high temperature, such trade‐offs appear to be common across taxa including seed beetles (Berger et al, 2017; Kim et al, 2020). An explanation for these patterns could be that females have evolved a “live‐fast die‐young” life‐history strategy to adapt to living in stressful adult environments. In such a strategy, we would expect females from stressful environments to invest more in early life reproduction at the cost of reduced late life reproduction and life spans due to trade‐offs between these life‐history components (Kirkwood and Rose, 1991; Stearns 1989), than females from favorable environments. Although we saw a similar reduction in adult life span of males from hot adult temperatures, whether this is due to life‐history trade‐offs, constraints, or an adaptation could be investigated by future studies.

It is also possible that the effects of adult temperature on patterns of female reproduction are not causal, but rather act through female mortality. For instance, if hot temperatures increased expected future mortality rates, this could lead to females terminally investing in early adult life reproduction (Clutton‐Brock, 1984). This increased early adult life investment would in turn lead to fewer eggs being available for laying later, which results in a less equal distribution of eggs over a female's life span, leading to faster senescence (e.g., Gribble et al, 2018).

### EVIDENCE FOR AN INTERACTION BETWEEN DEVELOPMENTAL AND ADULT ENVIRONMENTS

In general, there was very little indication of significant interactions between developmental and adult environments. However, we found that developmental and adult‐life environments interacted significantly to affect age‐dependent change in male weight. This interaction suggested that males that experienced hot temperature at both stages showed a significantly slower rate of age‐dependent weight loss than males that experienced favorable developmental but hot adult temperatures. This result is consistent with the beneficial acclimation hypothesis (Wilson and Franklin, 2002), which is a form of adaptive plasticity. On the other hand, these differences could also result from selective disappearance of poorer quality beetles during development stage in hot temperatures but not ancestral temperatures.

Previous studies looking at age‐dependent traits have not found any evidence for beneficial acclimation effects when manipulating foraging environments (Briga et al, 2019), or diet and temperature (Min et al, 2020). Future studies could test whether beneficial acclimation effects could actually be due to allocation of resources towards somatic (i.e., body weight) maintenance by males that experience developmental stress, at the expense of investment in reproduction. This is crucial because heat is known to affect rates of spermatogenesis and testis size in seed beetles (Vasudeva et al, 2014), and thus could create such environment‐dependent life‐history trade which would explain our results for male weight senescence.

There was no evidence that any other traits measured in our study were affected by an interaction between developmental and adult environments. This contrasts with recent studies in *Drosophila* (Duxbury and Chapman, 2020, Min et al, 2020), which found that female reproductive senescence was affected by an interaction between developmental and adult diets. One possible explanation as to why we did not find such interactions could be because seed beetles do not eat or drink during adulthood, whereas *Drosophila* do (Duxbury and Chapman, 2020; Min et al, 2020). This could allow species such as *Drosophila* to compensate for a poor developmental environment e.g., low nutrition and higher rates of heat‐induced desiccation by feeding and drinking more when they experience favorable environments in adulthood (Metcalfe and Monaghan, 2001). Thus, the effects of poor developmental environments would depend on the quality of adult environments, leading to an interaction between environments at different life stages for such species, although studies on facultative adult feeder/ capital breeders such as seed beetles are needed to allow us to make such comparisons. It is also possible that compensations in adulthood for stress experienced during developmental, are easier when diet rather than temperature is manipulated, something future studies could test.

### SEX‐SPECIFIC EFFECTS

In general, male and female traits responded in the same direction to both developmental and adult environments. Female emergence weight was more affected by developmental environments than was male emergence weight. On the other hand, male life span was more affected by adult environments than was female life span. A reason for these interactions between sex and emergence weight could be that females begin with heavier weights than males, and thus have more potential for change in their weight (as seen in Iglesias‐Carrasco et al., 2020). Additionally, females are heavier and have higher water content, thus heat‐induced desiccation during development could affect them more than it affects males. Alternatively, having a higher water content could also make females more resistant to desiccation than males.

For life span, because males had longer life spans to begin with, this trait could have a greater potential for change than female life spans. Although previous studies show seed beetle males on average live shorter lives than females (Berger et al, 2016), our results show the opposite. This is likely because females mated and laid eggs in our study, whereas males remained virgins and mating history has been shown to affect seed beetle life spans (den Hollander and Gwynne, 2009; Ronn et al, 2006; Sanghvi et al 2021).

A reason for sex‐specific responses to environmental change could be due to males and females having evolved to have different life history strategies (e.g., pace of life syndromes) under different adult environments (Hamalainen et al, 2018; Immonen et al, 2018). Testing for such sex × environment interactions would help us understand how male and female seed beetles (which are an invasive species in many countries) will respond to increasing temperatures caused by climate change, and whether warming would reduce the performance of one sex more than the other (Rogell et al, 2014).

### OTHER PATTERNS IN SENESCENCE

Finally, we also found two patterns in senescence that could have interesting ecological and evolutionary consequences. First, there were significant differences in rates of reproductive senescence between individual females (shown in, e.g., Bouwhuis et al, 2010) and between families (of full‐sibs). This was evidenced by models that allowed the slopes of both different females and different families to vary providing a better fit to the data than a model that did not have random effects of family and only allowed intercepts (but not slopes) of females to vary. The observation of between‐family differences could indicate (broad‐sense) heritability of reproductive senescence rates, although we note that variation between families could also be driven by nongenetic maternal effects. Further analysis including estimation of narrow‐sense heritability, accounting for maternal effects, would be needed to assess the potential for reproductive senescence rates to evolve in response to selection. Such a study would inform us whether climate warming would be able to select on senescence rates.

Second, we found evidence for selective disappearance of lighter males with increasing age. This may have masked individual‐level decreases in weight due to heavier individuals surviving longer. To our knowledge, such evidence of individual‐level patterns of senescence being masked by population‐level patterns of ageing due to selective disappearance of individuals has previously only been shown in vertebrates (Bouwhuis et al, 2009; Hayward et al, 2013).

## Conclusions

Ours is one of the first studies to test whether heat stress experienced at different life stages interacts to affect individual life histories and senescence. We show that depending on the trait and the sex measured, either developmental and/or adult environments can affect the resulting phenotype. Although these phenotypic differences could be due to adaptive plasticity of life‐history strategies under different environments, they could also be due to life‐history trade‐offs or due to temperature acting on these different traits in a noncausal manner. Overall, we show that the way environments affect an individual's phenotypic responses is complex. Considering that our findings suggest that the adult environment might have a stronger influence than developmental environments on traits that are not direct measures of reproduction, studies should integrate the effects of environments experienced during development and adulthood to avoid biased results, as well as measure a diverse range of life‐history traits. We show that environments can affect traits at different stages of ontogeny and that age‐dependent changes in traits depend on the effects of environment experienced at that age and on the past environments experienced. In particular, studies that test for the effects of changing temperatures must measure organisms throughout their lifetime to achieve a complete picture of how organisms will respond to climate change.

## AUTHOR CONTRIBUTIONS

KS, MLH, and LEBK designed the experiment. KS collected the data. KS and FZ analyzed the data. MIC, MLH, and KS wrote the manuscript. All authors contributed equally in critical assessment of the manuscript and subsequent revisions.

## CONFLICT OF INTEREST

The authors declare no conflict of interest.

## DATA ARCHIVING

Data and code for this study can be accessed from the Open Science Framework (https://osf.io/dt5ah/). Data and code have been uploaded on DRYAD (https://doi.org/10.5061/dryad.nvx0k6dvt).

Associate Editor: N. G. Prasad

Handling Editor: T. Chapman

## Supporting information


**Figure S1**: Effects of developmental temperature (DevT) and sex (M‐ males, F‐ females) on emergence weight (mg) of beetles. Significant interaction between DevT and sex seen in Table S5 arises because‐ male weight is less affected by developmental temperature compared to female weight; or because differences in male vs female weight are greater in ancestral developmental environments than in hot developmental environments.
**Figure S2**: Effects of adult temperature (AdultT) and sex (M‐ males, F‐ females) on adult life span (days) of beetles. Significant interaction seen in Table S6, between adult temperature and sex arises because‐ male life span is more affected by adult temperature than female life span is; or due to the difference in male vs female life spans being greater in ancestral adult environments than in hot adult environments.
**Figure S3**: Change in weight (mg) with adult age (in days), of males, with plot binned by male adult life span ((age range for each bin] = sample size of males in each bin). *Ancestral Developmental and Ancestral Adult (* ***AA*** *), Ancestral Developmental and Hot Adult (* ***AH*** *), Hot Developmental and Ancestral Adult (* ***HA***), *and Hot Developmental and Hot Adult (* ***HH***) temperatures. Each smoothed spline is created using the average weight of males in that given life span group. Males which have higher weights at adult age 0 (emergence) live longer than males which have lower emergence weights. 4 to 6 bins were created for each treatment because these allowed the clearest interpretation of curves visually, with the least amount of lines crossing over.
**Table S1**. Final sample sizes of emerged individuals used to analyse adult traits. *Ancestral Developmental and Ancestral Adult (* ***AA*** *), Ancestral Developmental and Hot Adult (* ***AH*** *), Hot Developmental and Hot Adult (* ***HH*** *), and Hot Developmental and Ancestral Adult (* ***HA***) temperatures.
**Table S3**. Effect of Developmental temperature on **emergence success**. Modelled using a GLMM logistical regression with binomial error distribution and “logit” link function. (N = 2303).
**Table S4**. The effect of developmental temperature and sex on **development time** of beetles (N = 1370) Modelled using LMMs. Power ((y^λ^ ‐1)/λ)) transformation of data (λ= −0.141414). “Full model” shows the parameter estimates and significance values for the model with two‐way interactions (highlighted in grey), while the main‐effects models shows parameter estimates and significance values for interpretation of only the main‐effects (highlighted in grey).
**Table S5**: The effect of developmental temperature and sex on **emergence weight** of beetles (N = 1369). Modelled using LMMs. “Full model” shows the parameter estimates and significance values for the model with two‐way interactions (highlighted in grey), while the main‐effects models shows parameter estimates and significance values for interpretation of only the main‐effects (highlighted in grey).
**Table S6**: The effect of developmental temperature, adult temperature, and sex on **adult lifespan** of beetles (N = 1324), after accounting for the effects of emergence weight. Modelled using LMMs. “Full model” shows the parameter estimates and significance values for the model with three‐way interactions (highlighted in grey), while the two‐way and main‐effects models shows parameter estimates and significance values for interpretation of only the two‐way interactions and main‐effects (highlighted in grey) respectively.
**Table S7**. The effects of developmental and adult temperature on **female fertility** (N = 635). Modelled using a GLMM (Logit link function). “Full model” shows the parameter estimates and significance values for the model with two‐way interactions (highlighted in grey), while the main‐effects model shows parameter estimates and significance values for interpretation of only the main‐effects (highlighted in grey).
**Table S8**: The effects of developmental and adult temperature on **female lifetime reproductive success (LRS**) after accounting for selective disappearance (N = 635). Modelled using a GLMM (link = log). “Full model” shows the parameter estimates and significance values for the model with two‐way interactions (highlighted in grey), while the main‐effects model shows parameter estimates and significance values for interpretation of only the main‐effects (highlighted in grey).
**Table S9**: The effects of developmental temperature, adult temperature, and sex on **age‐dependent mortality** (N = 1329). Modelled using a Cox‐ proportional hazards mixed effects model. “Full model” shows the parameter estimates and significance values for the model with three‐way interactions (highlighted in grey), while the two‐way and main‐effects model shows parameter estimates and significance values for interpretation of only the two‐way interactions and main‐effects (highlighted in grey) respectively.
**Table S10**: The effects of developmental temperature, adult temperature, and age on **age‐dependent** (**daily) fecundity of females** (N = 619). Modelled using GLMM (negative binomial error distribution). “Full model” shows the parameter estimates and significance values for the model with three‐way interactions (highlighted in grey), while the two‐way interactions model shows parameter estimates and significance values for interpretation of only the two‐way interactions (highlighted in grey). Main‐effects model not included because it does not allow age to interact with treatment, thus does not inform us about age‐dependent changes. Adult age modelled as a continuous variable.
**Table S11**: The effects of developmental temperature, adult temperature, and age on **male weight (mg)**. Modelled using LMMs. Three‐way interactions (in grey). “Full” model uses males from both hot and ancestral adult temperatures (N = 673).
**Table S12**: Exploratory test conducted on age‐dependent male weight, shown in model S11. The effects of developmental temperature on age‐dependent male weight are analysed for males who only experience hot adult temperatures, to compare AH (*Ancestral Developmental and Hot Adult*) and HH (*Hot Developmental and Hot Adult*) treatments. (N = 354).
