# Supplementary material for: Effects of developmental and adult environments on ageing
Source: Evolution. 2022 Jul 19;76(8):1868–82. doi: 10.1111/evo.14567 (PMC9543291; doi:10.1111/evo.14567)
Supplement: Supplementary file 1 — Figure S1: Effects of developmental temperature (DevT) and sex (M‐ males, F‐ females) on emergence weight (mg) of beetles. Significant interaction between DevT and sex seen in Table S5 arises because‐ male weight is less affected by developmental temperature compared to female weight; or because differences in male vs female weight are greater in ancestral developmental environments than in hot developmental environments. Figure S2: Effects of adult temperature (AdultT) and sex (M‐ males, F‐ females) on adult life span (days) of beetles. Significant interaction seen in Table S6, between adult temperature and sex arises because‐ male life span is more affected by adult temperature than female life span is; or due to the difference in male vs female life spans being greater in ancestral adult environments than in hot adult environments. Figure S3: Change in weight (mg) with adult age (in days), of males, with plot binned by male adult life span ((age range for each bin] = sample size of males in each bin). Ancestral Developmental and Ancestral Adult ( AA ), Ancestral Developmental and Hot Adult ( AH ), Hot Developmental and Ancestral Adult ( HA ), and Hot Developmental and Hot Adult ( HH ) temperatures. Each smoothed spline is created using the average weight of males in that given life span group. Males which have higher weights at adult age 0 (emergence) live longer than males which have lower emergence weights. 4 to 6 bins were created for each treatment because these allowed the clearest interpretation of curves visually, with the least amount of lines crossing over. Table S1. Final sample sizes of emerged individuals used to analyse adult traits. Ancestral Developmental and Ancestral Adult ( AA ), Ancestral Developmental and Hot Adult ( AH ), Hot Developmental and Hot Adult ( HH ), and Hot Developmental and Ancestral Adult ( HA ) temperatures. Table S3. Effect of Developmental temperature on emergence success. Modelled using a GLMM logistical regression with bino [file EVO-76-1868-s001.docx]

**Supplementary material**

**Contents**

1. Final sample sizes used to analyse adult traits (page 2)
2. Details of models used to analyse each trait (page 3)
3. Model outputs (page 5)
4. Note on selective disappearance of lighter males during adulthood (page 19)

Data and code related to this study can be found on Open Science Framework: https://osf.io/dt5ah/

1. **Final sample sizes used to analyse adult traits**

**Table S1.** Final sample sizes of emerged individuals used to analyse adult traits. *Ancestral Developmental and Ancestral Adult (****AA****), Ancestral Developmental and Hot Adult (****AH****), Hot Developmental and Hot Adult (****HH****), and Hot Developmental and Ancestral Adult (****HA****)* temperatures.

| **Block** | **Treatment** | **Female (N)** | **Male (N)** |
| --- | --- | --- | --- |
| **1**  (Total of 260 eggs setup in Hot Developmental temp. and 260 in Ancestral Developmental temp. from 26 families) | AA | 46 | 45 |
|  | AH | 53 | 42 |
|  | HH | 26 | 21 |
|  | HA | 25 | 21 |
|  | | | |
| **2**  (Total of 600 eggs setup in Hot Developmental temp. and 300 in Ancestral Developmental temp. from 30 families)* | AA | 62 | 55 |
|  | AH | 68 | 70 |
|  | HH | 101 | 120 |
|  | HA | 107 | 114 |
|  | | | |
| **3**  (Total of 600 eggs setup in Hot Developmental temp. and 300 in Ancestral Developmental temp. from 30 families)* | AA | 40 | 60 |
|  | AH | 50 | 68 |
|  | HH | 29 | 38 |
|  | HA | 28 | 35 |

*More beetles were set up in blocks 2 and 3 because it became apparent after running Block 1 that beetles had higher mortality in the Hot Developmental treatment than in the Ancestral developmental temperature.

1. **Details of models used to analyse each trait**

***Adult age-independent traits***

In general, we started with the most complex model (full) to analyse all traits. For age independent traits, this full model involved an interaction between developmental temperature, adult temperature, and sex, whenever three-way interactions were present, in order to only interpret three-way interactions between temperatures and sex. It also included block as a fixed effect, and beetle family as a random effect. In order to only interpret two-way interactions between developmental temperature, adult temperature, and sex, we further created two-way interaction models wherever two-way interactions were present. Additionally to only interpret main effects, we created a main-effects model. These model were used to interpret their respective effects irrespective of the significance of their P values.

*Emergence success:* We ran a GLMM with a binomial error distribution and “logit” link function. Our binomial response variable was whether a beetle emerged from an egg (“1”) or not (“0”). We included developmental temperature and block as fixed effects.

*Development time:* We created, a LMM in which we included developmental temperature and its interaction with sex, and block as fixed effects. Development times for beetles were power transformed using, prior to final analysis, due to non-normality of model residuals when using untransformed data. The *MASS* package and *boxcox* function obtains a lambda value, which was then added in the formula ((y^λ^ -1)/λ)) to obtain the most appropriate transformation for development time.

*Emergence weight:* We analysed emergence weight using an LMM in which we included developmental temperature and its interaction with sex, and block as fixed effects.

*Adult lifespan:* We modelled developmental temperature, adult temperature, and sex and their interactions as fixed effects using an LMM. We included emergence weight of beetles as a covariate in the models to ensure that the effects of temperature on adult lifespan was not a result of differences in emergence weight of beetles. The model which included emergence weight was a better fit to the data than one which excluded emergence weight (ΔAIC=68). We also included block as a fixed effect in the model.

*Fertility (Females only):* To analyse whether developmental and adult temperatures affected the likelihood that a female laid at least one egg in her lifetime (henceforth called fertility), we modelled developmental and adult temperature as well as their two-way interaction as a fixed effect, and our binomial response variable was whether a female laid at least one egg (“1”) or not (“0”) during her lifetime. We used the function *glm* in the package *stats* to model the data with a binomial error distribution and “logit” link function. The random effect of family explained no variation, thus was dropped from the model.

*Lifetime reproductive success (LRS):* We modelled the total number of eggs laid by females across her lifespan using a GLMM (poisson error distribution with log link function). Developmental and adult temperature as well as their two-way interaction were included as fixed effects. We added female adult lifespan as a covariate to account for selective disappearance (van de Pol & Verhulst, 2006). To correct for over-dispersion and non-convergence in this model, the function *glmerControl*(optimizer=”bobyqa”) in the package *lme4* and observation-level random effects were used.

***Adult age-dependent traits***

We analysed three age-dependent traits using daily (for female fecundity and beetle survival) and alternate day (for male weight) observations of adults following emergence. Because we wished to test how developmental and adult environments interacted with each other, and their effect on ageing rates, these models included three-way interactions between the two temperature treatments and age. In all age-dependant models, age was fit as a continuous variable. Our model building approach has been described in the main MS.

*Adult age-dependent mortality:* To test for the interactive effects of developmental temperature, adult temperature, and sex on age-dependent mortality we constructed a Cox mixed-effects proportional-hazards model using the function and package *coxme* (Therneau, 2014). This included developmental temperature, adult temperature, sex and their interactions as fixed effects and the adult lifespan of beetles as the response variable.

*Female age-dependent (daily) fecundit*y: To check whether there were any effects of developmental and adult temperatures on age-dependent number of eggs laid by females, we fitted a GLMM using the function *glmmTMB* (family=nbinom2) in the package *glmmTMB*, to account for zero-inflation of data. Eggs laid per day was the response variable. We included two separate three-way interactions of developmental temperature, adult temperature, and age (linear and quadratic) as fixed effects. The model with both linear and quadratic terms for age was a better fitting model than a model with just a linear term for age (ΔAIC= 123). We included a random effect of female ID to account for non-independence due to repeated measurements made on the same female. We also added adult lifespan as a fixed effect to account for selective disappearance.

In order to test for the presence of broad sense heritability for age-dependent female fecundity, we first created a model without the random effect of family (which only allowed the intercepts of different females to vary). We then compared this model to a model which allowed both slopes and intercepts of different families and different females to vary using a log-likelihood ratio test under a chi squared distribution. Because the model which allowed both female and family, slopes and intercepts to vary was a better fit to the data (ΔAIC= 414), this is the model presented.

*Adult age-dependent male weight:* To analyse the effects of developmental and adult temperatures on age-dependent weight of males, we used an LMM. The weight of adult males measured every second day, from emergence until death, was the response variable. In the model, we included two separate three-way interactions of developmental temperature, adult temperature, and age (one term for linear and one for quadratic effects of age) as fixed effects. There was a possibility that the age-dependent changes in weight of males depended on their emergence weight, such that males with lower emergence weights would show a shallower decline in weight. To account for this, we added a fixed effect of an interaction between male age and emergence weight in the model. The model with an interaction term between emergence weight and age was better fitting than the model without this term (ΔAIC= 2483, ΔDF= 2). Additionally, the model with a quadratic term for age was a better fitting model than a model which did not include quadratic effects of age (ΔAIC= 2442, ΔDF= 4). We included a random effect of beetle ID to account for non-independence due to repeated measurements of weight on the same males. We also added a covariate of adult lifespan to account for selective disappearance of males.

Because the three-way interaction between developmental and adult temperature and age was significant in this model, we explored these effects further to test for whether this was due to a beneficial acclimation effect, in males who experienced hot adult temperatures. Specifically, we tested whether males who experienced hot temperatures during both development and adulthood, lost weight at a slower rate than males who experienced hot temperatures during adulthood but not during development. To do this, the model only used data from males who experienced hot adult temperatures, to compare males in AH (ancestral developmental and hot adult) vs HH (hot developmental and hot adult) treatments.

1. **Model Outputs**

**(**Note: for all tables, significant P values highlighted in bold.). DevT= Developmental temperature, AdultT= Adult temperature, Adult age= Adult age, N= number of beetles included in the analysis. Three-way interaction, two-way interaction, and main-effects models only used to interpret three-way interactions, two-way interactions, and main-effects respectively (Shaded in grey).

**Table S3.** Effect of Developmental temperature on **emergence success**. Modelled using a GLMM logistical regression with binomial error distribution and “logit” link function. (N = 2303)

| **Fixed effect** | **Estimate** | **SE** | **z** | **P** |
| --- | --- | --- | --- | --- |
| (Intercept) | 1.092 | 0.141 | 7.750 | 0.000 |
| DevT (Hot) | -1.657 | 0.112 | -14.853 | **<0.001** |
| Block2 | 1.731 | 0.177 | 9.754 | <0.001 |
| Block3 | -0.435 | 0.166 | -2.619 | 0.009 |
|  |  |  |  |  |
| **Random effect** | **Variance** | **SD** |  |  |
| Family | 0.173 | 0.416 |  |  |

**Table S4.** The effect of developmental temperature and sex on **development time** of beetles (N= 1370) Modelled using LMMs. Power ((y^λ^ -1)/λ)) transformation of data (λ= -0.141414). “Full model” shows the parameter estimates and significance values for the model with two-way interactions (highlighted in grey), while the main-effects models shows parameter estimates and significance values for interpretation of only the main-effects (highlighted in grey).

| **Full model** |  |  |  |  |  |
| --- | --- | --- | --- | --- | --- |
| **Fixed effect** | **Estimate** | **SE** | **DF** | **t** | **P** |
| (Intercept) | 2.854 | 0.005 | 186.600 | 576.192 | <0.001 |
| DevT (Hot) | -0.286 | 0.004 | 1331.000 | -67.099 | <0.001 |
| Sex (Male) | -0.008 | 0.004 | 1350.000 | -1.871 | 0.062 |
| Block 2 | -0.042 | 0.006 | 95.270 | -7.428 | 0.000 |
| Block 3 | 0.019 | 0.006 | 114.700 | 3.217 | 0.002 |
| DevT (Hot) * Sex (Male) | -0.004 | 0.006 | 1337.000 | -0.731 | 0.465 |
|  |  |  |  |  |  |
| **Random effects** | **Variance** | **SD** |  |  |  |
| Family | 0.000 | 0.015 |  |  |  |

| **Main-effects model** |  |  |  |  |  |
| --- | --- | --- | --- | --- | --- |
| **Fixed effect** | **Estimate** | **SE** | **DF** | **t** | **P** |
| (Intercept) | 2.855 | 0.005 | 156.000 | 605.464 | <0.001 |
| Block 2 | -0.042 | 0.006 | 95.300 | -7.433 | 0.000 |
| Block 3 | 0.019 | 0.006 | 114.700 | 3.226 | 0.002 |
| DevT (Hot) | -0.288 | 0.003 | 1337.000 | -94.522 | **<0.001** |
| Sex (Male) | -0.010 | 0.003 | 1343.000 | -3.421 | **0.001** |
|  |  |  |  |  |  |
| **Random effects** | **Variance** | **SD** |  |  |  |
| Family | 0.000 | 0.015 |  |  |  |

**Table S5**: The effect of developmental temperature and sex on **emergence weight** of beetles (N= 1369). Modelled using LMMs. “Full model” shows the parameter estimates and significance values for the model with two-way interactions (highlighted in grey), while the main-effects models shows parameter estimates and significance values for interpretation of only the main-effects (highlighted in grey).

| **Full model** |  |  |  |  |  |
| --- | --- | --- | --- | --- | --- |
| **Fixed effect** | **Estimate** | **SE** | **DF** | **t** | **P** |
| (Intercept) | 5.420 | 0.059 | 157.947 | 91.727 | <0.001 |
| DevT (Hot) | -1.094 | 0.047 | 1321.679 | -23.145 | <0.001 |
| Sex (Male) | -2.061 | 0.047 | 1340.556 | -43.926 | <0.001 |
| Block 2 | 0.431 | 0.069 | 88.533 | 6.205 | <0.001 |
| Block 3 | 0.596 | 0.073 | 103.857 | 8.186 | <0.001 |
| DevT (Hot) * Sex (Male) | 0.829 | 0.065 | 1327.921 | 12.710 | **<0.001** |
|  |  |  |  |  |  |
| **Random effect** | **Variance** | **SD** |  |  |  |
| Family | 0.041 | 0.202 |  |  |  |

| **Main-effects model** |  |  |  |  |  |
| --- | --- | --- | --- | --- | --- |
| **Fixed effect** | **Estimate** | **SE** | **DF** | **t** | **P** |
| (Intercept) | 5.206 | 0.058 | 138.966 | 89.616 | <0.001 |
| DevT (Hot) | -0.674 | 0.036 | 1328.723 | -18.837 | **<0.001** |
| Sex (Male) | -1.633 | 0.035 | 1334.524 | -47.110 | **<0.001** |
| Block 2 | 0.436 | 0.071 | 88.528 | 6.179 | <0.001 |
| Block 3 | 0.584 | 0.074 | 104.950 | 7.858 | <0.001 |
|  |  |  |  |  |  |
|  |  |  |  |  |  |
| **Random effect** | **Variance** | **SD** |  |  |  |
| Family | 0.040 | 0.200 |  |  |  |


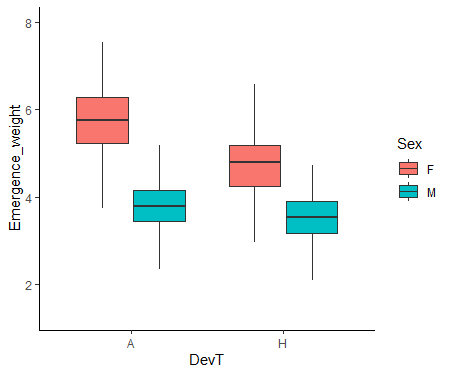


Figure S1: Effects of developmental temperature (DevT) and sex (M- males, F- females) on emergence weight (mg) of beetles. Significant interaction between DevT and sex seen in Table S5 arises because- male weight is less affected by developmental temperature compared to female weight; or because differences in male vs female weight are greater in ancestral developmental environments than in hot developmental environments.

**Table S6:** The effect of developmental temperature, adult temperature, and sex on **adult lifespan** of beetles (N=1324), after accounting for the effects of emergence weight. Modelled using LMMs. “Full model” shows the parameter estimates and significance values for the model with three-way interactions (highlighted in grey), while the two-way and main-effects models shows parameter estimates and significance values for interpretation of only the two-way interactions and main-effects (highlighted in grey) respectively.

| **Full model** |  |  |  |  |  |
| --- | --- | --- | --- | --- | --- |
| **Fixed effect** | **Estimate** | **SE** | **DF** | **t** | **P** |
| (Intercept) | 5.040 | 0.926 | 1172.221 | 5.446 | <0.001 |
| Emergence_weight | 1.333 | 0.157 | 1300.807 | 8.487 | <0.001 |
| Block 2 | 0.706 | 0.354 | 100.915 | 1.997 | 0.049 |
| Block 3 | 1.151 | 0.380 | 127.465 | 3.028 | 0.003 |
| DevT (Hot) | 1.420 | 0.427 | 1286.409 | 3.329 | 0.001 |
| AdultT (Hot) | -7.336 | 0.381 | 1256.661 | -19.267 | <0.001 |
| Sex (Male) | 10.566 | 0.505 | 1312.226 | 20.908 | <0.001 |
| DevT (Hot) * AdultT (Hot) | -0.215 | 0.539 | 1255.586 | -0.399 | 0.690 |
| DevT (Hot) * Sex (Male) | -0.245 | 0.556 | 1281.927 | -0.440 | 0.660 |
| AdultT (Hot) * Sex (Male) | -5.255 | 0.530 | 1253.394 | -9.922 | <0.001 |
| DevT (Hot) * AdultT (Hot)  * Sex (Male) | -0.516 | 0.747 | 1253.809 | -0.691 | 0.490 |
|  |  |  |  |  |  |
| **Random effect** | **Variance** | **SD** |  |  |  |
| Family | 0.806 | 0.898 |  |  |  |

| **Two-way interaction model** |  |  |  |  |  |
| --- | --- | --- | --- | --- | --- |
| **Fixed effect** | **Estimate** | **SE** | **DF** | **t** | **P** |
| (Intercept) | 4.968 | 0.919 | 1168.755 | 5.404 | <0.001 |
| Emergence_weight | 1.333 | 0.157 | 1301.650 | 8.488 | <0.001 |
| Block 2 | 0.709 | 0.353 | 101.040 | 2.006 | 0.048 |
| Block 3 | 1.152 | 0.380 | 127.664 | 3.034 | 0.003 |
| DevT (Hot) | 1.558 | 0.377 | 1291.811 | 4.137 | <0.001 |
| AdultT (Hot) | -7.201 | 0.327 | 1256.385 | -21.995 | <0.001 |
| Sex (Male) | 10.705 | 0.464 | 1313.965 | 23.089 | <0.001 |
| DevT (Hot) * AdultT (Hot) | -0.484 | 0.373 | 1254.105 | -1.297 | 0.195 |
| DevT (Hot) * Sex (Male) | -0.512 | 0.399 | 1298.507 | -1.283 | 0.200 |
| AdultT (Hot) * Sex (Male) | -5.515 | 0.373 | 1251.810 | -14.781 | **<0.001** |
|  |  |  |  |  |  |
| **Random effect** | **Variance** | **SD** |  |  |  |
| Family | 0.804 | 0.897 |  |  |  |

| **Main-effects model** |  |  |  |  |  |
| --- | --- | --- | --- | --- | --- |
| **Fixed effect** | **Estimate** | **SE** | **DF** | **t** | **P** |
| (Intercept) | 6.853 | 0.890 | 1141.142 | 7.698 | <0.001 |
| Emergence_weight | 1.316 | 0.160 | 1298.528 | 8.239 | **<0.001** |
| Block 2 | 0.641 | 0.357 | 104.855 | 1.794 | 0.076 |
| Block 3 | 1.150 | 0.386 | 134.113 | 2.983 | 0.003 |
| DevT (Hot) | 1.018 | 0.237 | 1304.599 | 4.300 | **<0.001** |
| AdultT (Hot) | -10.322 | 0.202 | 1259.304 | -51.097 | **<0.001** |
| Sex (Male) | 7.560 | 0.333 | 1312.574 | 22.727 | **<0.001** |
|  |  |  |  |  |  |
| **Random effect** | **Variance** | **SD** |  |  |  |
| Family | 0.691 | 0.831 |  |  |  |


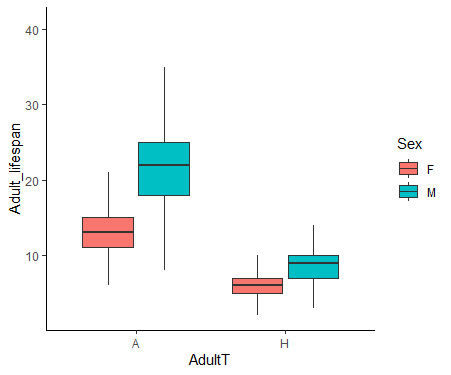


Figure S2: Effects of adult temperature (AdultT) and sex (M- males, F- females) on adult lifespan (days) of beetles. Significant interaction seen in Table S6, between adult temperature and sex arises because- male lifespan is more affected by adult temperature than female lifespan is; or due to the difference in male vs female lifespans being greater in ancestral adult environments than in hot adult environments.

**Table S7.** The effects of developmental and adult temperature on **female fertility** (N=635). Modelled using a GLMM (Logit link function). “Full model” shows the parameter estimates and significance values for the model with two-way interactions (highlighted in grey), while the main-effects model shows parameter estimates and significance values for interpretation of only the main-effects (highlighted in grey).

| **Full model** | **Fixed effects** | **Estimate** | **SE** | **z** | **P** |
| --- | --- | --- | --- | --- | --- |
|  | (Intercept) | 19.212 | 833.519 | 0.023 | 0.982 |
|  | DevT (Hot) | -18.073 | 833.519 | -0.022 | 0.983 |
|  | AdultT (Hot) | -14.980 | 833.519 | -0.018 | 0.986 |
|  | Block 2 | 1.822 | 0.413 | 4.413 | <0.001 |
|  | Block 3 | -0.417 | 0.395 | -1.057 | 0.290 |
|  | DevT (Hot)*AdultT (Hot) | 14.544 | 833.519 | 0.017 | 0.986 |
|  |  |  |  |  |  |
| **Main-effects model** | **Fixed effects** | **Estimate** | **SE** | **z** | **P** |
|  | (Intercept) | 5.158 | 0.779 | 6.621 | <0.001 |
|  | Block 2 | 1.825 | 0.413 | 4.413 | <0.001 |
|  | Block 3 | -0.421 | 0.395 | -1.066 | 0.287 |
|  | DevT (Hot) | -3.976 | 0.735 | -5.405 | **<0.001** |
|  | AdultT (Hot) | -0.511 | 0.328 | -1.557 | 0.119 |

**Table S8**: The effects of developmental and adult temperature on **female lifetime reproductive success (LRS**) after accounting for selective disappearance (N=635). Modelled using a GLMM (link= log). “Full model” shows the parameter estimates and significance values for the model with two-way interactions (highlighted in grey), while the main-effects model shows parameter estimates and significance values for interpretation of only the main-effects (highlighted in grey).

| **Full model** |  |  |  |  |
| --- | --- | --- | --- | --- |
| **Fixed effect** | **Estimate** | **SE** | **z** | **P** |
| (Intercept) | 5.013 | 0.159 | 31.450 | <0.001 |
| Adult_lifespan | -0.099 | 0.010 | -9.775 | <0.001 |
| Block2 | 0.994 | 0.108 | 9.198 | <0.001 |
| Block3 | 0.079 | 0.124 | 0.640 | 0.522 |
| DevT (Hot) | -1.102 | 0.094 | -11.699 | <0.001 |
| AdultT (Hot) | -0.802 | 0.098 | -8.174 | <0.001 |
| DevT (Hot)* AdultT (Hot) | -0.190 | 0.116 | -1.637 | 0.102 |
|  |  |  |  |  |
| **Random effect** | **Variance** | **SD** |  |  |
| Observation-level | 0.828 | 0.910 |  |  |
| Family | 0.031 | 0.175 |  |  |
|  |  |  |  |  |
| **Two-way interaction model** |  |  |  |  |
| **Fixed effect** | **Estimate** | **SE** | **z** | **P** |
| (Intercept) | 5.049 | 0.157 | 32.095 | <0.001 |
| Adult_lifespan | -0.098 | 0.010 | -9.746 | <0.001 |
| Block2 | 0.993 | 0.108 | 9.186 | <0.001 |
| Block3 | 0.079 | 0.123 | 0.643 | 0.520 |
| DevT (Hot) | -1.197 | 0.074 | -16.223 | **<0.001** |
| AdultT (Hot) | -0.882 | 0.085 | -10.366 | **<0.001** |
|  |  |  |  |  |
| **Random effect** | **Variance** | **SD** |  |  |
| Observation-level | 0.819 | 0.905 |  |  |
| Family | 0.032 | 0.179 |  |  |

**Table S9:** The effects of developmental temperature, adult temperature, and sex on **age-dependent mortality** (N = 1329). Modelled using a Cox- proportional hazards mixed effects model. “Full model” shows the parameter estimates and significance values for the model with three-way interactions (highlighted in grey), while the two-way and main-effects model shows parameter estimates and significance values for interpretation of only the two-way interactions and main-effects (highlighted in grey) respectively.

| **Full model** |  |  |  |  |  |
| --- | --- | --- | --- | --- | --- |
| **Fixed effects** | **coef** | **exp** | **SE** | **z** | **P** |
| Sex (Male) | -1.832 | 0.160 | 0.133 | -13.740 | <0.001 |
| AdultT (Hot) | 3.179 | 24.021 | 0.138 | 23.090 | <0.001 |
| DevT (Hot) | -0.373 | 0.688 | 0.121 | -3.080 | 0.002 |
| Block2 | -0.379 | 0.685 | 0.112 | -3.380 | 0.001 |
| Block3 | -0.498 | 0.608 | 0.119 | -4.190 | <0.001 |
| Sex (Male) * AdultT (Hot) | 0.405 | 1.500 | 0.175 | 2.320 | 0.020 |
| Sex (Male) * DevT (Hot) | 0.232 | 1.261 | 0.167 | 1.390 | 0.160 |
| AdultT (Hot) * DevT (Hot) | 0.287 | 1.332 | 0.167 | 1.710 | 0.087 |
| Sex (Male) * AdultT (Hot)  * DevT (Hot) | -0.296 | 0.744 | 0.228 | -1.300 | 0.190 |
|  |  |  |  |  |  |
| **Random effect** | **SD** | **Variance** |  |  |  |
| Family | 0.311 | 0.097 |  |  |  |
|  |  |  |  |  |  |
|  |  |  |  |  |  |
| **Two-way interaction  model** |  |  |  |  |  |
| **Fixed effects** | **coef** | **exp** | **SE** | **z** | **P** |
| Sex (Male) | -1.749 | 0.174 | 0.117 | -14.940 | <0.001 |
| AdultT (Hot) | 3.248 | 25.741 | 0.127 | 25.580 | <0.001 |
| DevT (Hot) | -0.292 | 0.747 | 0.104 | -2.810 | 0.005 |
| Block2 | -0.376 | 0.687 | 0.112 | -3.360 | 0.001 |
| Block3 | -0.495 | 0.610 | 0.119 | -4.170 | <0.001 |
| Sex (Male) * AdultT (Hot) | 0.254 | 1.290 | 0.130 | 1.950 | 0.051 |
| Sex (Male) * DevT (Hot) | 0.078 | 1.081 | 0.117 | 0.660 | 0.510 |
| AdultT (Hot) * DevT (Hot) | 0.128 | 1.137 | 0.114 | 1.120 | 0.260 |
|  |  |  |  |  |  |
| **Random effect** | **SD** | **Variance** |  |  |  |
| Family | 0.311 | 0.097 |  |  |  |
|  |  |  |  |  |  |
|  |  |  |  |  |  |
| **Main-effects model** |  |  |  |  |  |
| **Fixed effects** | **coef** | **exp** | **SE** | **z** | **P** |
| Block2 | -0.364 | 0.695 | 0.111 | -3.280 | 0.001 |
| Block3 | -0.494 | 0.610 | 0.118 | -4.170 | <0.001 |
| Sex (Male) | -1.565 | 0.209 | 0.066 | -23.780 | **<0.001** |
| AdultT (Hot) | 3.420 | 30.576 | 0.098 | 35.000 | **<0.001** |
| DevT (Hot) | -0.179 | 0.836 | 0.059 | -3.030 | **0.002** |
|  |  |  |  |  |  |
| **Random effect** | **SD** | **Variance** |  |  |  |
| Family | 0.311 | 0.097 |  |  |  |

**Table S10:** The effects of developmental temperature, adult temperature, and age on **age-dependent** (**daily) fecundity of females** (N = 619). Modelled using GLMM (negative binomial error distribution). “Full model” shows the parameter estimates and significance values for the model with three-way interactions (highlighted in grey), while the two-way interactions model shows parameter estimates and significance values for interpretation of only the two-way interactions (highlighted in grey). Main-effects model not included because it does not allow age to interact with treatment, thus does not inform us about age-dependent changes. Adult age modelled as a continuous variable.

Only the model which allowed both intercepts and slopes of different females and families to vary is presented, this being the best fit model that one without a random effect of family.

| **Full model** |  |  |  |  |
| --- | --- | --- | --- | --- |
| **Fixed effects** | **Estimate** | **SE** | **z** | **P** |
| (Intercept) | 3.722 | 0.229 | 16.273 | <0.001 |
| Block2 | 1.102 | 0.117 | 9.433 | <0.001 |
| Block3 | 0.214 | 0.133 | 1.607 | 0.108 |
| Adult_lifespan | -0.131 | 0.014 | -9.289 | <0.001 |
| DevT (Hot) | -0.656 | 0.171 | -3.840 | <0.001 |
| AdultT (Hot) | 0.289 | 0.202 | 1.430 | 0.153 |
| Adult age | -0.003 | 0.027 | -0.113 | 0.910 |
| Adult age^2 | -0.021 | 0.002 | -12.115 | <0.001 |
| DevT (Hot) * AdultT (Hot) | -0.237 | 0.258 | -0.919 | 0.358 |
| DevT (Hot) * Adult age | -0.265 | 0.040 | -6.670 | <0.001 |
| AdultT (Hot) * Adult age | -0.486 | 0.067 | -7.225 | <0.001 |
| DevT (Hot) * Adult age^2 | 0.017 | 0.003 | 6.541 | <0.001 |
| AdultT (Hot) * Adult age^2 | 0.012 | 0.009 | 1.395 | 0.163 |
| DevT (Hot) * AdultT (Hot)  * Adult age | 0.010 | 0.105 | 0.097 | 0.923 |
| DevT (Hot) * AdultT (Hot)  * Adult age^2 | 0.003 | 0.014 | 0.200 | 0.841 |
|  |  |  |  |  |
| **Random effect** | **Name** | **Variance** | **SD** | **Corr** |
| ID | (Intercept) | 1.390 | 1.179 |  |
|  | Adult age | 0.029 | 0.171 | -0.740 |
| Family | (Intercept) | 0.072 | 0.268 |  |
|  | Adult age | 0.003 | 0.055 | -0.540 |
|  |  |  |  |  |
| **Two-way interaction model** |  |  |  |  |
| **Fixed effects** | **Estimate** | **SE** | **z** | **P** |
| (Intercept) | 3.790 | 0.221 | 17.133 | <0.001 |
| Block2 | 1.101 | 0.117 | 9.425 | <0.001 |
| Block3 | 0.216 | 0.133 | 1.622 | 0.105 |
| Adult_lifespan | -0.131 | 0.014 | -9.305 | <0.001 |
| DevT (Hot) | -0.790 | 0.127 | -6.207 | <0.001 |
| AdultT (Hot) | 0.174 | 0.165 | 1.054 | 0.292 |
| Adult age | -0.010 | 0.025 | -0.415 | 0.678 |
| Adult age^2 | -0.021 | 0.002 | -12.381 | <0.001 |
| DevT (Hot) * Adult age | -0.249 | 0.033 | -7.514 | **<0.001** |
| AdultT (Hot) * Adult age | -0.479 | 0.053 | -8.988 | **<0.001** |
| DevT (Hot) * Adult age^2 | 0.017 | 0.002 | 6.856 | **<0.001** |
| AdultT (Hot) * Adult age^2 | 0.013 | 0.007 | 1.800 | 0.072 |
|  |  |  |  |  |
| **Random effect** | **Name** | **Variance** | **SD** | **Corr** |
| ID | (Intercept) | 1.389 | 1.179 |  |
|  | Adult age | 0.029 | 0.171 | -0.740 |
| Family | (Intercept) | 0.074 | 0.271 |  |
|  | Adult age | 0.003 | 0.055 | -0.550 |

**Table S11:** The effects of developmental temperature, adult temperature, and age on **male weight (mg)**. Modelled using LMMs. Three-way interactions (in grey). “Full” model uses males from both hot and ancestral adult temperatures (N=673).

| \| **Model** \| **Fixed effects** \| **Estimate** \| **SE** \| **DF** \| **t** \| **P** \| \| --- \| --- \| --- \| --- \| --- \| --- \| --- \| \| **Full** \| (Intercept) \| -0.038 \| 0.042 \| 983.4 \| -0.896 \| 0.37 \| \|  \| Block2 \| 0.177 \| 0.019 \| 94.26 \| 9.551 \| <0.001 \| \|  \| Block3 \| 0.247 \| 0.019 \| 109.2 \| 12.707 \| <0.001 \| \|  \| Adult lifespan \| 0.02 \| 0.001 \| 740 \| 16.28 \| <0.001 \| \|  \| Adult age \| -0.056 \| 0.003 \| 5097 \| -19.385 \| <0.001 \| \|  \| Emergence.weight \| 0.837 \| 0.011 \| 1055 \| 78.47 \| <0.001 \| \|  \| DevT(Hot) \| -0.041 \| 0.017 \| 1357 \| -2.445 \| 0.015 \| \|  \| AdultT(Hot) \| 0.297 \| 0.023 \| 1335 \| 12.976 \| <0.001 \| \|  \| Adult age^2 \| 0.002 \| 0 \| 4726 \| 34.532 \| <0.001 \| \|  \| Adult age*Emergence.weight \| -0.017 \| 0.001 \| 5187 \| -26.469 \| <0.001 \| \|  \| DevT(Hot)*AdultT(Hot) \| -0.013 \| 0.024 \| 1991 \| -0.528 \| 0.598 \| \|  \| DevT(Hot)*Adult age \| 0.017 \| 0.002 \| 4707 \| 8.333 \| <0.001 \| \|  \| AdultT(Hot)*Adult age \| -0.205 \| 0.005 \| 4767 \| -41.096 \| <0.001 \| \|  \| DevT(Hot)*Adult age^2 \| -0.001 \| 0 \| 4757 \| -6.867 \| <0.001 \| \|  \| AdultT(Hot)*Adult age^2 \| 0.011 \| 0.001 \| 4900 \| 20.91 \| <0.001 \| \|  \| DevT(Hot)*AdultT(Hot) *Adult age \| 0.036 \| 0.007 \| 4745 \| 5.1 \| **<0.001** \| \|  \| DevT(Hot)*AdultT(Hot) *Adult age^2 \| -0.002 \| 0.001 \| 4872 \| -2.831 \| **0.005** \| | | | | | | | |  |  |  |  |  |  |
| --- | --- | --- | --- | --- | --- | --- | --- | --- | --- | --- | --- | --- | --- | --- | --- | --- | --- | --- | --- | --- | --- | --- | --- | --- | --- | --- | --- | --- | --- | --- | --- | --- | --- | --- | --- | --- | --- | --- | --- | --- | --- | --- | --- | --- | --- | --- | --- | --- | --- | --- | --- | --- | --- | --- | --- | --- | --- | --- | --- | --- | --- | --- | --- | --- | --- | --- | --- | --- | --- | --- | --- | --- | --- | --- | --- | --- | --- | --- | --- | --- | --- | --- | --- | --- | --- | --- | --- | --- | --- | --- | --- | --- | --- | --- | --- | --- | --- | --- | --- | --- | --- | --- | --- | --- | --- | --- | --- | --- | --- | --- | --- | --- | --- | --- | --- | --- | --- | --- | --- | --- | --- | --- | --- | --- | --- | --- | --- | --- | --- | --- | --- | --- | --- | --- | --- | --- | --- | --- | --- |
|  | **Random effect** | **Variance** | **SD** |  |  |  |  |  |  |  |  |  |  |
|  | ID | 0.011 | 0.107 |  |  |  |  |  |  |  |  |  |  |
|  | Family | 0.002 | 0.044 |  |  |  |  |  |  |  |  |  |  |

**Table S12:** Exploratory test conducted on age-dependent male weight, shown in model S11. The effects of developmental temperature on age-dependent male weight are analysed for males who only experience hot adult temperatures, to compare AH (*Ancestral Developmental and Hot Adult*) and HH (*Hot Developmental and Hot Adult*) treatments. (N=354)

| **Fixed effects** | **Estimate** | **SE** | **DF** | **t** | **P** |
| --- | --- | --- | --- | --- | --- |
| (Intercept) | -0.117 | 0.054 | 847.900 | -2.189 | 0.029 |
| Adult age | -0.180 | 0.010 | 1468.000 | -17.501 | <0.001 |
| Emergence weight | 0.883 | 0.015 | 830.800 | 60.551 | <0.001 |
| DevT (Hot) | -0.054 | 0.017 | 999.200 | -3.119 | 0.002 |
| Adult age^2 | 0.013 | 0.001 | 1425.000 | 24.618 | <0.001 |
| Block 2 | 0.153 | 0.020 | 83.070 | 7.678 | <0.001 |
| Block 3 | 0.206 | 0.021 | 92.310 | 9.934 | <0.001 |
| Adult lifespan | 0.047 | 0.003 | 347.900 | 15.869 | <0.001 |
| Adult age*Emergence weight | -0.039 | 0.002 | 1463.000 | -15.708 | <0.001 |
| DevT (Hot)*Adult age | 0.049 | 0.007 | 1368.000 | 7.011 | **<0.001** |
| DevT (Hot)*Adult age^2 | -0.003 | 0.001 | 1447.000 | -3.596 | **<0.001** |
|  |  |  |  |  |  |
| **Random effects** | **Variance** | **SD** |  |  |  |
| ID | 0.005 | 0.071 |  |  |  |
| Family | 0.001 | 0.039 |  |  |  |

1. **Note on selective disappearance of lighter males during adulthood**

To further examine whether the effects of an increase in average weight seen towards the end of life in males was due to selective disappearance of lighter males, we visualised individual-level changes in weight with age. We binned males into categories based on their lifespans to plot their age-dependent weight as smoothed splines (Figure S3). This showed a strong correlation of males of a lower emergence weight having shorter lifespans.

Figures S3 shows that there is a decline in weight with age for each curve in each treatment, and that heavier individuals live longer, thus any increase in weight with advancing age, as seen in Figure 6, and due to a quadratic function of age in Table S11, can be attributed to selective disappearance of lighter beetles rather than an actual increase in weight of individuals with age.

Similar to the results seen below for male weight, which show that late-adult life increases the average value of a trait due to selective disappearance, we think that the increases in female fecundity seen during late-adult lifespan are also due to the same reason, i.e. selective disappearance.


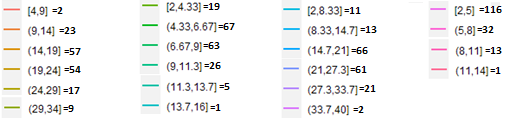

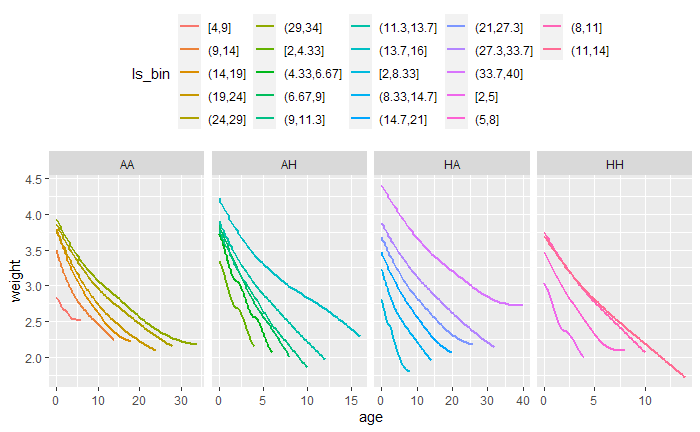


Figure S3: Change in weight (mg) with adult age (in days), of males, with plot binned by male Adult lifespan ((age range for each bin] = sample size of males in each bin). *Ancestral Developmental and Ancestral Adult (****AA****), Ancestral Developmental and Hot Adult (****AH****), Hot Developmental and Ancestral Adult (****HA****)*, *and Hot Developmental and Hot Adult (****HH****)* temperatures*.* Each smoothed spline is created using the average weight of males in that given lifespan group. Males which have higher weights at adult age 0 (emergence) live longer than males which have lower emergence weights. 4 to 6 bins were created for each treatment because these allowed the clearest interpretation of curves visually, with the least amount of lines crossing over.
